# The Regenerative Plasticity of Isolated Urodele Myofibers and Its Dependence on *Msx1*

**DOI:** 10.1371/journal.pbio.0020218

**Published:** 2004-08-17

**Authors:** Anoop Kumar, Cristiana P Velloso, Yutaka Imokawa, Jeremy P Brockes

**Affiliations:** **1**Department of Biochemistry and Molecular Biology, University College LondonLondonUnited Kingdom; **2**Department of Anatomy and Developmental Biology, Royal Free and University College Medical SchoolLondonUnited Kingdom; **3**Center for Developmental Biology, Laboratory for Evolutionary RegenerationRIKEN, Chuo-ku, KobeJapan

## Abstract

The conversion of multinucleate postmitotic muscle fibers to dividing mononucleate progeny cells (cellularisation) occurs during limb regeneration in salamanders, but the cellular events and molecular regulation underlying this remarkable process are not understood. The homeobox gene *Msx1* has been studied as an antagonist of muscle differentiation, and its expression in cultured mouse myotubes induces about 5% of the cells to undergo cellularisation and viable fragmentation, but its relevance for the endogenous programme of salamander regeneration is unknown. We dissociated muscle fibers from the limb of larval salamanders and plated them in culture. Most of the fibers were activated by dissociation to mobilise their nuclei and undergo cellularisation or breakage into viable multinucleate fragments. This was followed by microinjection of a lineage tracer into single fibers and analysis of the labelled progeny cells, as well as by time-lapse microscopy. The fibers showing morphological plasticity selectively expressed *Msx1* mRNA and protein. The uptake of morpholino antisense oligonucleotides directed to *Msx1* led to a specific decrease in expression of Msx1 protein in myonuclei and marked inhibition of cellularisation and fragmentation. Myofibers of the salamander respond to dissociation by activation of an endogenous programme of cellularisation and fragmentation. Lineage tracing demonstrates that cycling mononucleate progeny cells are derived from a single myofiber. The induction of *Msx1* expression is required to activate this programme. Our understanding of the regulation of plasticity in postmitotic salamander cells should inform strategies to promote regeneration in other contexts.

## Abstract 2

Amphibians such as the salamander can regenerate their limbs. This paper explores how multinucleate muscle cells transform into mononuclear cells and begin to proliferate during regeneration

## Introduction

There is currently a significant focus on strategies to promote regeneration in adult mammals and therefore a renewed interest in the mechanisms that underlie regeneration in urodele amphibians. An adult salamander such as the newt or axolotl can regenerate its limbs and tail, jaws, ocular tissues such as the lens, and small sections of the heart (Goss 1969; Eguchi et al. 1974; Oberpriller and Oberpriller 1974; Okada 1991; Ghosh et al. 1994; Brockes 1997; Nye et al. 2003). A key feature of urodele regeneration is the local plasticity of differentiated cells at the site of tissue injury or removal (Brockes and Kumar 2002; Odelberg 2002; Del Rio-Tsonis and Tsonis 2003; Tanaka 2003). This has been investigated for pigment epithelial cells of the iris (Eguchi et al. 1974; Simon and Brockes 2002; Imokawa and Brockes 2003; Imokawa et al. 2004), cardiomyocytes (Oberpriller et al. 1995; Bettencourt-Dias et al. 2003), and skeletal myofibers and myotubes (Hay 1959; Lo et al. 1993; Tanaka et al. 1997, 1999; Kumar et al. 2000; Echeverri et al. 2001), all of which reenter the cell cycle during regeneration, in contrast to their mammalian counterparts. A second aspect of plasticity is the ability of multinucleate skeletal muscle cells to fragment into viable mononucleate cells that then contribute to the regenerate. This process, sometimes referred to as cellularisation, was described in classical studies of limb regeneration (Thornton 1938; Hay 1959), but was first analysed with marked cells by implantation of cultured newt myotubes labelled by microinjection with a lineage tracer (Lo et al. 1993) or by an integrated retrovirus (Kumar et al. 2000). The myotubes were effectively converted to mononucleate cells that proliferated in the blastema, and this process occurred in cells that were blocked from cell cycle reentry (Velloso et al. 2000), thus showing that the two aspects of plasticity are not linked mechanistically. In an important recent contribution, myofibers were labelled in situ by microinjection in the tail of the larval axolotl (Echeverri et al. 2001). After amputation of the tail, the myofibers fragmented into viable mononucleate cells, thus establishing that cellularisation occurs during regeneration and contributes to the proliferative zone or blastema.

Our understanding of this intriguing process has received considerable impetus from the recognition of two manipulations that induce mammalian myotubes to undergo fragmentation. The first is exposure to myoseverin, a trisubstituted purine derivative isolated from a combinatorial library (Rosania et al. 2000). It evokes depolymerisation of microtubules, apparently by interacting directly with tubulin, as well as inducing changes in the expression of genes that are implicated in tissue remodelling and wound healing. The second is the conditional expression of the homeobox gene *Msx1* in mouse myotubes (Odelberg et al. 2000). *Msx1* has been studied as an antagonist of myogenic and osteogenic differentiation (reviewed in Bendall and Abate-Shen 2000) and is expressed in the migrating precursor cells of limb muscle during chick development, apparently to prevent precocious differentiation (Bendall et al. 1999). The expression in mouse C2C12 myotubes evokes two aspects of plasticity that occur in 5%–10% of the cells; the first is cleavage of the cells into smaller multinucleated myotubes, which remain viable, and the other is the formation of mononucleate cells capable of division (Odelberg et al. 2000). In the latter case, the clonal progeny of a single myotube were shown to be capable of several pathways of mesenchymal differentiation.

The studies on cellularisation by myoseverin and *Msx1* have underlined that mammalian as well as urodele cells are capable of this response (Rosania et al. 2000; Odelberg 2002). *Msx1* is expressed during urodele limb regeneration (Carlson et al. 1998; Koshiba et al. 1998), as well as during fin (Akimenko et al. 1995; Nechiporuk and Keating 2002) and heart regeneration in the zebrafish (Raya et al. 2003), along with other *Msx* family genes. Therefore, it becomes important to investigate whether it controls cellularisation during regeneration. Although prior studies of this process have used the multinucleate myotube as the target cell in culture, the critical target during epimorphic regeneration is the more differentiated striated myofiber. The regeneration of muscle fibers in vertebrates proceeds by the mobilisation of reserve satellite cells (Chargé and Rudnicki 2004), and these have been described in myofibers of larval salamander limbs (Popiela 1976). Their participation in cellularisation was excluded in the earlier experiments on urodele cells by selective injection of a lineage tracer into myotubes in culture (Lo et al. 1993) or myofibers in the salamander tail (Echeverri et al. 2001). In order to address these various questions, we have established a culture system in which striated myofibers are dissociated from the limb of larval salamanders and attach to a culture substrate where they can be observed by time-lapse microscopy. The fibers are found to be activated by dissociation to undergo cellularisation and viable fragmentation, and this depends on expression of *Msx1*.

## Results

### Dissociated Myofibers in Culture

**Figure 1:**
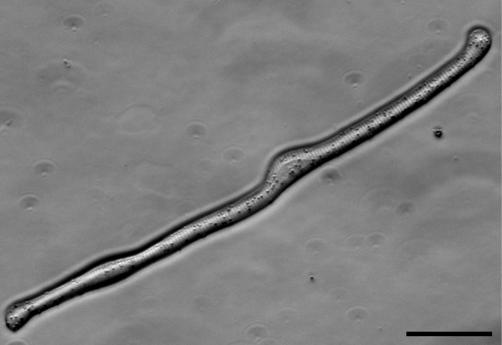
A Live Striated Myofiber from the Larval Salamander Photomicrograph of a live striated myofiber dissociated from the larval limb musculature and adhering to the culture dish in serum-free medium. This cell has the appearance of a normal quiescent fiber and was photographed with VAREL optics at 48 h after plating. Scale bar, 50 μm.

In order to obtain striated myofibers, tissue was isolated from the limbs of two species of larval salamander (Ambystoma maculatum or Ambystoma mexicanum), which have been used interchangeably with comparable results. After removing the epidermis, the tissue was dissociated by proteolytic digestion, filtered through a sieve to remove most of the mononucleate cells, and plated in serum-free medium. The striated myofibers readily attached to the culture dish (Figure 1; Figure S1) and were found to express myosin heavy chain (MHC) and titin after antibody staining. In view of the potential contribution of satellite type cells to the issues under investigation, cultures were treated with a viable nuclear stain and the myofibers were examined carefully at 2 d after plating. Of 1,290 fibers examined in seven independent cultures, there were only 46 examples of mononucleate cells adherent to myofibers, and such cells were not observed in the cases of plasticity that are discussed here.

When cultures were labelled with tritiated thymidine, no labelled nuclei were observed in multinucleate cells after labelling for 24 h (540 myofibers, five cultures) or 48 h (263 myofibers, three cultures), while 16% of the mononucleate cells were labelled in the latter case. It is noteworthy that the absence of S-phase entry in nuclei within multinucleate cells includes the population of myofibers that undergoes the events of cellularisation or fragmentation described below.

### Cellularisation of Myofibers after Implantation

**Figure 2:**
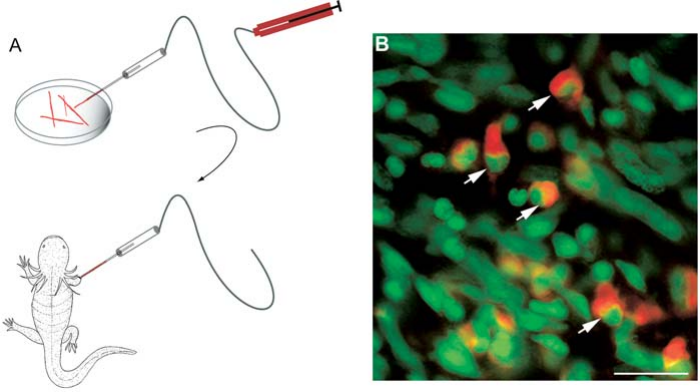
Cellularisation of Striated Myofibers after Implantation into a Larval Limb Blastema (A) Schematic diagram of procedure. After dissociation of larval limb musculature, the cells were loaded with a cell tracker dye and single myofibers taken up into a suction micropipette, prior to injection into a larval limb blastema as detailed in the Materials and Methods. (B) Section of a limb at 48 h after implantation of CellTracker Orange-labelled myofibers. The section has been counterstained with the nuclear stain Sytox green. Note the dye-labelled mononucleate cells (arrowed). Scale bar, 20 μm.

The dissociation of viable myofibers has allowed us to evaluate their plasticity after implantation into a limb blastema, a procedure that has previously been performed only on myotubes formed in cell culture. Fibers were labelled with a cell tracker dye in suspension after dissociation, and single fibers were examined to verify the absence of any adherent mononucleate cells and were drawn into a glass micropipette (Figure 2A). A few fibers (see Materials and Methods) were injected from the pipette into the early forelimb regenerate of a larval axolotl. The regenerating limbs were sectioned 2–4 d later, and many examples were observed of mononucleate cells labelled with the tracker dye (Figure 2B). Such cells were clearly mononucleates, as determined by analysis of serial sections, and were observed in 17 out of 23 animals implanted with labelled myofibers. We conclude that these cells readily undergo cellularisation in the environment of the limb blastema.

### Plasticity in Culture

After 48 h in culture, some of the striated fibers remained viable, but showed no significant change in morphology and retained the appearance of the cell shown in Figure 1. The remainder of the fibers showed various changes in morphology, and these were investigated either by microinjection of single fibers with the lineage tracer Texas red (TR)–dextran and subsequent analysis of the progeny cells or by sequential digital time-lapse observation.

#### Cellularisation.

**Figure 3:**
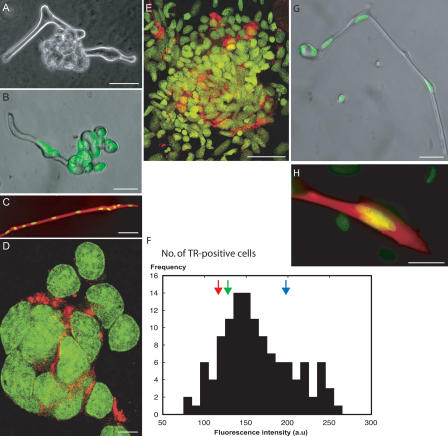
Plasticity of Isolated Myofibers (A) Phase-contrast micrograph of a live cell at 3 d after plating, showing a lobulated structure in the middle of the fiber. (B) Micrograph of a live fiber at 2 d after plating, showing budding of nuclei at one end. The cell has been counterstained with Syto 13. (C) Fluorescence micrograph of a myofiber at 24 h after microinjection with TR–dextran. The cell has been counterstained with Syto 13 dye to show the nuclei. (D) Fluorescence micrograph of a colony formed from a single myofiber injected 24 h earlier with TR–dextran. The cell has flattened on the substrate and the nuclei are stained with Syto 13 dye. (E) Fluorescence micrograph of a colony formed from the progeny of several myofibers in proximity that were injected 5 d earlier with TR–dextran. (F) Analysis of the DNA content of cells derived from myofibers injected 5 d earlier with TR–dextran. The DNA content was determined by image analysis of the nuclei of mononucleate TR-positive cells that had been stained with Hoechst (see Materials and Methods). The green arrow is the value for G_0_ nuclei in quiescent myofibers, while the blue arrow is the G_2_/M value determined for mononucleate cells with anti-phosphohistone H3. The red arrow is the G_1_ value determined for mononucleate cells. (G) Photomicrograph of a live myofiber, 48 h after plating, showing a binucleate bud formed at the end. The cell was stained as for (B). (H) Fluorescence micrograph of a bud containing three nuclei stained with Syto13 (yellow) derived from a myofiber that contained at least five nuclei and that was injected with TR–dextran (red). Scale bars: (B), (C), and (G), 100 μm; (A), (E), and (H), 50 μm; and (D), 10 μm.

Approximately 10% of the total population of myofibers underwent changes in nuclear localisation within the cells such that a lobulated or ‘cauliflower’ structure formed in the middle or end of the cell (Figure 3A and 3B). This occurred without labelling by tritiated thymidine or any participation by adherent mononucleate cells, which were rarely present on such fibers. The lobules, each of which contained a nucleus, were rapidly resolved into adherent mononucleate cells. In order to analyse these events, single myofibers were microinjected with TR–dextran so as to fill the cells with tracer (Figure 3C). We employed the 70 kDa dextran, which is not transferred across gap junctions (Coelho and Kosher 1991; Landesman et al. 2000). In cases in which fibers formed the cauliflower structure and underwent cellularisation, the mononucleate cells in the initial colony were labelled with the tracer in a rim of cytoplasm around the nucleus (Figure 3D). In some cases, adjacent fibers were injected and the progeny of the myofibers gave rise after 5–7 d to overlapping dense colonies with many labelled cells (Figure 3E). These cells did not express detectable levels of MHC after staining by indirect immunofluorescence.

We have analysed the DNA content of single Hoechst-stained nuclei by normalised measurements of fluorescence intensity in TR-labelled cells within such colonies, and an example of a representative distribution for a single colony is shown in Figure 3F. This also shows the corresponding values (shown by arrows in Figure 3F) for G_0_ nuclei in myofibers, G_1_ nuclei in mononucleate cells, and G_2_/M nuclei in mononucleate cells labelled with antibody to phosphohistone H3. The histogram of DNA content for cells in the colony is comparable to that previously observed for cycling newt mononucleate cells (Tanaka et al. 1997). The relatively long S-phase in urodele cells leads to a prominent contribution of cells with DNA content between 2N and 4N. In addition, there were examples of TR-labelled mononucleate cells in M-phase, as determined with anti-phosphohistone H3. We conclude that the progeny mononucleate cells are able to traverse S-phase and enter mitosis after cellularisation.

#### Fragmentation.

**Figure 4:**
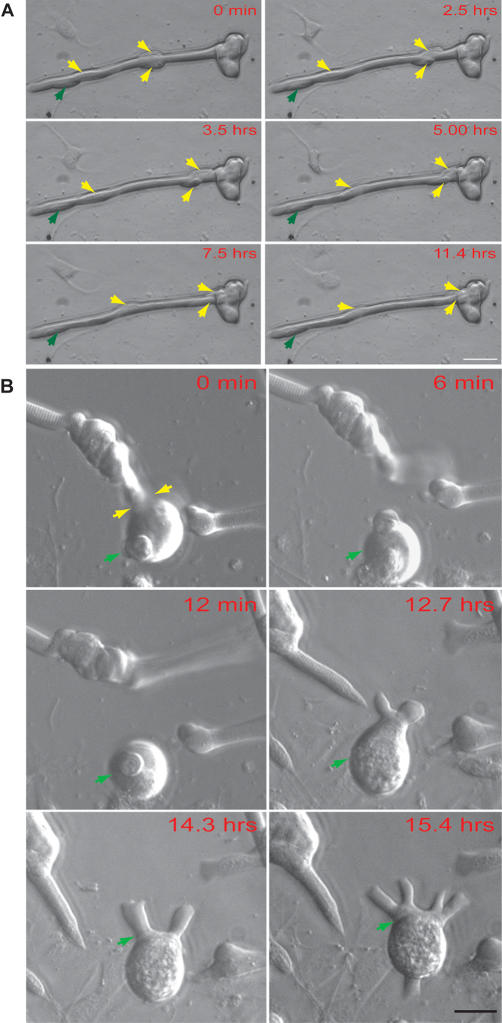
Analysis of Nuclear Migration and Fragmentation by Time-Lapse Microscopy (A) Single frames illustrating the migration of three nuclei (yellow arrows) along a myofiber, of which two are incorporated into a terminal aggregate by 11.4 h. One nucleus (green arrow) remained stationary during this period. (B) Single frames illustrating the production of viable multinucleate fragments from a myofiber. Note the presence of a trinucleate aggregate (arrowed green) that separates after lateral breakage of the fiber (0 min, arrowed yellow). This fragment subsequently extends cytoplasmic processes (14.3 and 15.4 h) and migrates over the culture substratum. Series (A) and (B) begin at 6 h after plating. Scale bars: (A) 50 μm; (B) 200 μm.

In a second aspect of plasticity, which was shown by 40%–70% of the total population of myofibers, the initial stages also involved the migration of nuclei to form local aggregates, often of two or three nuclei, within the fiber. The migration of nuclei into a terminal aggregate is illustrated by selected images from a time-lapse video analysis (Figure 4A; Video S1). The series of Figure 4B illustrates a trinucleate terminal aggregate that fragments from the body of the myofiber (yellow arrows). This fragment remained adherent and extended cytoplasmic processes. In some cases, the nuclear aggregate formed a bud that was discharged into the medium. An example of a multinucleate bud formed at the end of a fiber is shown in Figure 3G. In cases in which fibers containing at least five nuclei had been injected with TR–dextran, such buds were often observed to adhere as viable bi- or trinucleate-labelled cells (see Figure 3H). The multinucleate progeny resulting from these processes did not label with tritiated thymidine or undergo division.

#### Inhibition by taxol.

**Figure 5:**
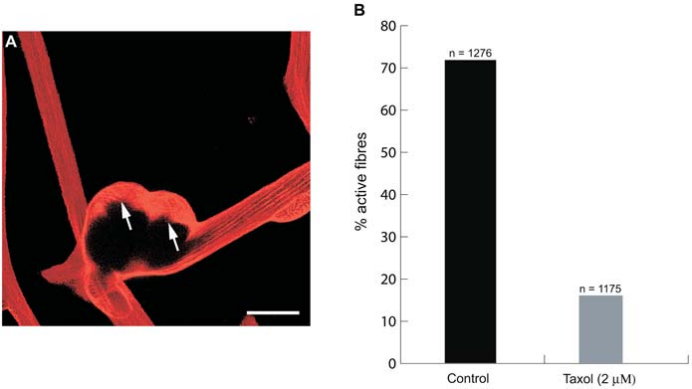
Plasticity and Microtubule Depolymerization (A) The distribution of microtubules surrounding a multinucleate aggregate on a myofiber, as analysed by staining with anti-β-tubulin. Note the relatively disordered state of the tubulin (arrowed) in the vicinity of the nuclei. The fiber was stained at 48 h after plating. Scale bar, 50 μm. (B) Taxol inhibits the activation of myofibers after dissociation. Myofibers were dissociated and cultured in the presence of taxol as described in the Materials and Methods. The number of active fibers was determined as described.

In view of the evidence that implicates microtubules as a target for myoseverin, we stained the cultures with antibody to β-tubulin. Although tubulin was polymerised in microtubules parallel to the axis of the fibers, the regions of nuclear aggregation were associated with depolymerised tubulin (Figure 5A). In order to assess the functional relevance of depolymerisation, we exposed the cultures to 2 μM taxol, an agent that stabilises microtubules and inhibits division of mononucleate urodele cells without effects on cell viability or adhesion of myofibers to the culture substrate. Whereas 80% of the control fibers showed the morphologies associated with plasticity, as described above (see Materials and Methods for the criteria), only 16% were observed in the case treated with taxol (Figure 5B), although the total number of adherent cells was unaffected. This suggests that localised depolymerisation of microtubules may be a significant target for the regulation of these responses.

### Expression of *Msx1*

**Figure 6:**
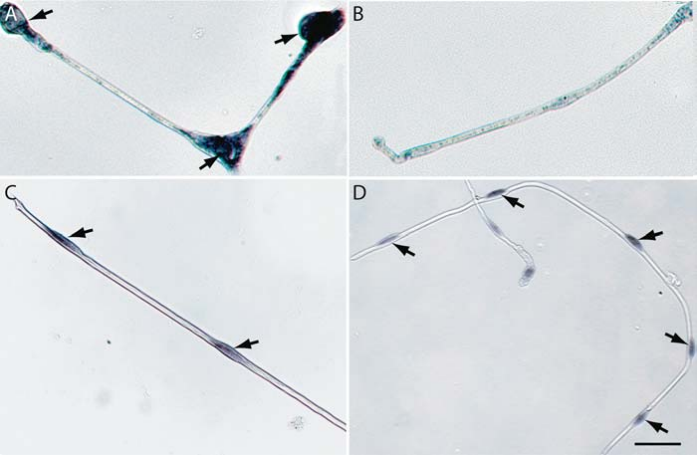
Analysis of mRNA Expression in Myofibers at 48 h by In Situ Hybridisation (A) Expression of *Msx1* mRNA in active myofiber. Note the accumulation of reaction product around nuclear aggregates (arrowed). (B) Absence of significant *Msx1* mRNA expression in a quiescent fiber. This image is taken from the same culture as (A). (C) Expression of *NRad* mRNA in nuclei of quiescent fibers (arrowed). Comparable intensity was observed for *NRad* expression by active fibers. (D) Expression of *Msx1* mRNA in nuclei of fibers (arrowed) made quiescent by culture in taxol. Note the difference in *Msx1* expression levels between the taxol-induced inactive fibers and normal quiescent myofibers in (B). Scale bar, 50 μm.

The cultures were reacted with a digoxygenin-substituted antisense riboprobe to axolotl *Msx1*. The mononucleate cells and inactive myofibers showed little or no reactivity, but active fibers showed strong reactivity with the probe in the vicinity of nuclear aggregations (Figure 6A and 6B). Several control probes were negative on both classes of fibers, while the quiescent fibers as well as the active ones were reactive to antisense probes to urodele *EF1a* and *Nrad* (Figure 6C). In view of the relationship between *Msx1* expression and plasticity in mouse myotubes (Odelberg et al. 2000), the expression in the active fibers is suggestive of a role in the endogenous urodele programme.

In an initial investigation of this possibility, cultures were arrested as before by treatment with taxol, followed by reaction with the *Msx1* antisense probe. In parallel control cultures, only 4.2% of the inactive fibers (*n* = 404) showed any reaction with the probe, whereas 51% of all myofibers (*n* = 820) showed reactivity. After treatment with taxol, 56.3% of inhibited fibers (*n* = 765) were positive, whereas 63% of all myofibers (*n* = 901) showed expression of *Msx1*. It is clear, therefore, that the arrest of nuclear mobilisation does not prevent the early expression of *Msx1* in activated myofibers. These data are consistent with an upstream role for *Msx1* in the activation of plasticity, but direct evidence for functional activity has come from antisense perturbation.

### Activity of Morpholino-Substituted Oligonucleotides

**Figure 7:**
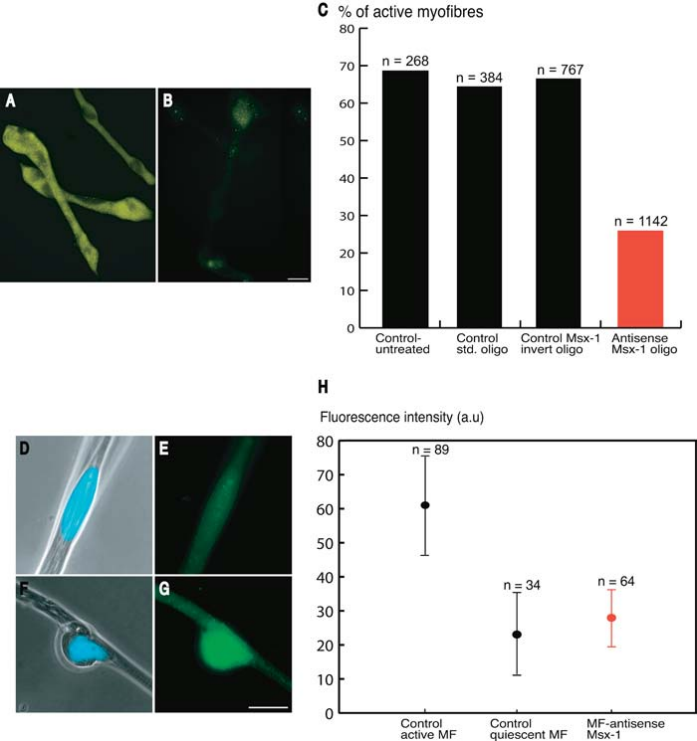
Analysis of the Functional Role of Msx1 Expression by Exposure to Morpholino Antisense Oligonucleotides (A and B) Uptake of morpholino by myofibers. Myofibers were dissociated in the presence of biotinylated (A) or control (B) morpholinos and analysed by tyramide signal amplification at 24 h after plating. Note the positive signal in (A), dependent on the presence of biotin moiety. In three different experiments 70%–90% of the fibers were loaded as determined with this assay. Scale bar, 50 μm. (C) Functional effect of loading various morpholinos. Note that loading *Msx1* antisense leads to a specific decrease in the proportion of active fibers relative to controls. (D–G) Staining of myofibers with antibody to Msx1 protein. (D and E) Fluorescence micrograph of a nucleus in a quiescent myofiber stained with Hoechst for DNA (D) and Msx1 protein (E). (F and G) Fluorescence micrograph of a nucleus in an active myofiber stained for DNA (F) and Msx1 protein (G). These images (D–G) were taken from the same culture. Scale bar, 20 μm. (H) Distribution of fluorescence intensity of nuclei in myofibers after staining with antibody to Msx1. The distributions for control active fibers and control quiescent fibers were determined for cells in the same culture and are significantly different (ANOVA, *p* < 0.001 at 95% confidence level). The distribution for antisense-treated quiescent fibers is not significantly different from that for control quiescent fibers.

In order to evaluate the uptake of morpholino-substituted oligonucleotides, larval myofibers were dissociated as usual in the presence of 10 μM biotinylated morpholinos or underivatised morpholinos. The cells were cultured for 48 h and then analysed by a detection procedure involving tyramide signal amplification (see Materials and Methods). Approximately 70%–90% of the fibers showed uptake of the biotinylated oligonucleotides in different experiments (Figure 7A), and no signal was detectable in the absence of oligonucleotide or with underivatised morpholinos (Figure 7B). The cells are thus effectively loaded by dissociation in the presence of morpholinos.

Limb tissue was dissociated in the presence of control morpholinos or a morpholino antisense reagent directed at the translation initiation sequence of axolotl *Msx1*. The resulting cultures were analysed in parallel for the proportion of active fibers and the antisense reagent reproducibly and specifically decreased this by 60%–70% (Figure 7C). This led, as expected from the mechanism of such reagents, to the presence of inhibited fibers that expressed *Msx1* mRNA after in situ hybridisation, and the proportion of such *Msx1* positive and inhibited cells was increased by 5-fold relative to parallel cultures incubated with control oligonucleotides.

The myofiber cultures were stained by indirect immunofluorescence with a rabbit antibody to Msx1 in order to evaluate the level of expression of the homeoprotein in the nuclei. There was a significant difference is staining of nuclei between active and quiescent fibers in the same culture (Figure 7D–7G). The level of expression in nuclei of different fibers was estimated by quantitative image analysis, and the distribution of intensities is shown in Figure 7H. There was a significant difference in the fluorescence intensity of nuclei in active and quiescent fibers in control cultures (Figure 7H), consistent with the difference in mRNA levels observed by in situ hybridisation (see Figure 6A and 6B). The distribution of intensities for nuclei in quiescent fibers in parallel cultures treated with antisense oligonucleotides to Msx1 was not significantly different from the control quiescent distribution (Figure 7H). It should be noted that more than half of the quiescent fibers were inhibited as a result of the antisense treatment, thus indicating that the antisense distribution reflects a significant decrease in protein expression in the nucleus relative to active fibers. We conclude that expression of a critical level of Msx1 protein is necessary for the fibers to exhibit plasticity in this culture system.

## Discussion

The plasticity of isolated urodele myofibers as described here has not been observed in previous work on dissociated mouse myofibers (Rosenblatt et al. 1995; Blaveri et al. 1999). These apparently retain their morphological identity in culture without undergoing viable fragmentation or cellularisation. In preliminary work on myofibers dissociated from the forelimb of *Xenopus* tadpoles (stages 56–63), we have observed fragmentation comparable to that described here for fibers of the larval salamander, but no cellularisation. It is possible, therefore, that there is a gradation in the degree of plasticity after dissociation, and this may be related to the ability to undergo reversal during regeneration, although more work is required to investigate these comparative issues. It is interesting that the mononucleate progeny of cellularisation were observed to reenter the cell cycle, while multinucleate fragments retained the postmitotic arrest of the parental fibers. At least half of the salamander fibers were activated after dissociation and could be scored by morphological criteria as an index of plasticity, as well as by analysis of gene expression in situ. The occurrence of cellularisation did not reflect the activation of adherent mononucleate cells since the injection of a nontransferable tracer into the fibers resulted in labelling of the mononucleate progeny, and furthermore the mobilisation of nuclear aggregates occurred without any detectable S-phase reentry. It is probable that the process of enzymatic and mechanical dissociation mimics the activation events after amputation, either in terms of mechanical factors sensed by the fibers or the release of signals from the tissue or matrix. Earlier experiments on microinjected fibers in the larval tail have explored the stimuli required to trigger cellularisation and concluded that activation apparently required both ‘clipping’ at the end of the fiber as well as tissue injury in the vicinity (Echeverri et al. 2001). It has also been reported that crude extracts from early regenerates of the adult newt limb are able to induce cellularisation of newt and mouse myotubes in culture (McGann et al. 2001). The precise nature of the signal(s) that couples tissue injury to activation of this response remains an important subject for future investigation, particularly as it may be a key difference between urodeles and mammals.

One striking consequence of fiber activation is the appearance of the *Msx1* transcript, and our work strongly supports the hypothesis that *Msx1* is a pivotal regulator of plasticity in differentiated cells. Although taxol treatment is able to block the internal reorganisation in activated fibers, it does not inhibit the induction of *Msx1*, suggesting that microtubule depolymerisation, while being a direct target of myoseverin (Rosania et al. 2000), may also be a downstream target for regulation by *Msx1*. The striated myofibers are more highly differentiated than the newt A1 myotubes employed for implantation or the C2C12 mouse myotubes used to assay myoseverin and *Msx1*. The events of cellularisation, cleavage, or budding off from myofibers are preceded by migration of nuclei to generate local concentrations, reminiscent of the events leading to formation of the neuromuscular junction (Merlie and Sanes 1985; Englander and Rubin 1987), although mouse myotubes seem to undergo lateral breakage without such reorganisation (Rosania et al. 2000). This migration is inhibited by taxol, and nuclear migration in other contexts is dependent on microtubule function (Morris 2003). All of the events described for the myofibers occur without entry into S-phase, as determined previously for cellularisation of myotubes after implantation (Velloso et al. 2001). The formation of mononucleate cells is followed by rapid division and loss of myosin expression, and these cells are presumably the culture equivalent of muscle-derived blastemal cells.

The activity of the *Msx1* gene has recently been implicated in digit tip regeneration in fetal and neonatal mice by comparing regeneration in normal and *Msx1* mutant animals (Reginelli et al. 1995; Han et al. 2003). It has also been shown that transgenic expression of an activated Msx1 protein can induce tail regeneration in larval *Xenopus* during the refractory period between stages 45 and 47 (Beck et al. 2003). This evidence, taken in conjunction with the present study and that of Odelberg et al. (2000), indicates that this gene is an important regulator of regeneration. Various activities have been associated with the protein, including a role as a repressor of transcription (reviewed in Bendall and Abate-Shen 2000), for example, of various myogenic differentiation genes in C2C12 myotubes (Odelberg et al. 2000) and also as a positive regulator of genes that promote cell cycling such as cyclin D (Hu et al. 2001). Our analysis of the myofiber cultures provides evidence for its ability to mobilise a postmitotic cell, for example, by nuclear migration and cellularisation, without S-phase reentry in the syncytium, and this suggests a different aspect of its activity as a regulator. Studies on mammalian myotubes should continue to be informative, while the present system, with its ready incorporation of antisense oligonucleotides, should be helpful for relating such studies to the endogenous programme of urodele regeneration. This in turn should assist the long-term goal of promoting the reversal of cellular differentiation as a strategy for mammalian regeneration (Chargé and Rudnicki 2004).

## Materials and Methods

### 

#### Tissue dissociation and culture of myofibers

The forelimbs and hind limbs of the larval spotted salamander (A. maculatum) or axolotl (A. mexicanum) (3–5 cm size) were removed, the epidermis was peeled off, and the tissue was rinsed in serum-free amphibian MEM (AMEM) (Ferretti and Brockes 1988) prior to dissociation for 3 h at 26 °C in PBS containing 0.15% collagenase (Type 1A, Sigma, St. Louis, Missouri, United States), 0.8% Dispase II (Roche, Basel, Switzerland), 0.15% crystalline bovine serum albumin, 0.3% D-glucose, and 0.15 mg/ml DNase I (Roche). After 30 min of incubation, the tissues were gently triturated through a fire-polished glass pasteur pipette to aid the detachment of myofibers from the bone. After incubation, the suspension was triturated several times, centrifuged at 400 rpm for 10 min, resuspended in AMEM, and filtered through a 35 μm sieve (VWR International, Poole, United Kingdom) to remove most of the mononucleate cells. The retentate was rinsed with AMEM and plated on 35 mm Falcon Primaria (Becton-Dickinson, Palo Alto, California, United States) tissue culture dishes. Cultures were maintained at 25 °C with 2.5% CO_2_ in a humidified incubator as described elsewhere (Ferretti and Brockes 1988). After attachment of the myofibers to the culture dish by overnight incubation, the culture media was replaced with serum-free AMEM or AMEM supplemented with 10% foetal bovine serum.

#### Labelling and implantation of myofibers

Myofibers were dissociated as above, retained in suspension in a sterile bacteriological dish (Bibby Sterilin, Stone, United Kingdom), and incubated with 10 μM CellTracker Orange CMTMR (Molecular Probes, Eugene, Oregon, United States) for 30 min at 25 °C. The labelling was terminated by addition of 10% AMEM, and the cells were incubated for 45 min at 25 °C to permit enzymatic activation of the dye. The cell suspension was diluted several fold to allow observation of myofibers at low density. The forelimbs of axolotl larvae (7–10 cm size) were amputated at mid humerus level under tricaine (0.1%) anaesthesia 48 h before injection of labelled myofibers (see Figure 2A). The animals were anaesthetized, and the forelimbs were positioned under a stereo zoom microscope. The myofiber suspension was placed under inverted microscope, and the myofibers were drawn into a glass micropipette (30 μm tip diameter) using an oil-driven manual microinjector (Sutter Instruments, Novato, California, United States) mounted on a Narishige (Tokyo, Japan) MMO-1 micromanipulator. The skin was punctured with a tungsten needle in order to introduce the blunt end of the micropipette. Three to eight myofibers were picked, examined carefully to verify the absence of any adherent mononucleate cells, and injected into each limb regenerate. Contralateral limbs were mock injected with medium from the suspension. The regenerates were removed at 48 h and 96 h after injection, fixed in 4% paraformaldehyde (PFA), and processed (Kumar et al. 2000). Serial longitudinal sections of 60 μm were cut on a cryostat (Leica, Solms, Germany), air dried, dehydrated in PBS, and counterstained with 2.5 μM Sytox Green (Molecular Probes). The sections were observed under epifluorescence on an Axiophot microscope (Zeiss, Jena, Germany).

#### Microinjection of cultured myofibers with conjugated dextran

Myofibers were incubated in AMEM containing 2,3-butanedionne monoxime (BDM) (4 mM) for 30 min to prevent contraction of the myofibers (Bettencourt-Dias et al. 2003) and maintained in the same medium during microinjection. The culture dishes were placed under a Zeiss Axiovert microscope and microinjected with TR-conjugated dextran (TR–dextran, 70 kDa; Molecular Probes). The medium was changed immediately after injection and the cultures were returned to the incubator. To identify the labelled myofibers and their mononucleate progeny, cultures were counterstained in Syto13 (200 nM; Molecular Probes) live nucleic acid stain for 30 min and observed under fluorescence microscope with a dual band pass (FITC/TRITC) filter.

#### Live imaging of myofiber plasticity

To record the coordinates of the myofibers in culture, the dish was scored underneath with a scalpel, and cells in each grid square were observed daily and images were acquired with a CCD camera (Sony, Tokyo, Japan). For time-lapse microscopy, myofiber cultures were placed under an Axiovert microscope fitted with an incubation chamber maintained at 26 °C and 3% CO_2_, and phase contrast or variable relief contrast (VAREL) (Zeiss) images were acquired using a digital camera controlled through Image-Pro Plus software (Media Cybernetics, Silver Spring, Maryland, United States). A sequence gallery was created using Image Pro-Plus and images of interest were selected, digitally enhanced, and processed in Adobe Photoshop 6.0 (Adobe, San Jose, California, United States).

#### [^3^H]thymidine labelling.

Myofibers were incubated in 1 μCi/ml [^3^H] thymidine (Amersham Biosciences, Little Chalfont, United Kingdom) for 24 h, fixed in 1% glutaraldehyde, and processed for autoradiography (Velloso et al. 2000).

#### DNA cytometry

DNA content in myofiber nuclei and TR–dextran-labelled mononucleate progeny was measured quantitatively after fixation and staining of the nuclei with Hoechst 33258 (2 μg/ml; Sigma). Baseline values for nuclear DNA content in cycling axolotl mononucleate cells were measured in parallel after incorporation of 5-deoxy-2′-bromouridine (BrdU) (1 μM; see Tanaka et al. 1997). The BrdU-labelled cells were processed for double immunofluorescence with monoclonal antibody against BrdU and rabbit antibody to anti-phosphohistone H3 (Velloso et al. 2000; Bettencourt-Dias et al. 2003). The nuclei were counterstained with Hoechst dye as above. All images were acquired using 12-bit cooled CCD camera (Photonic Sciences, Robertsbridge, United Kingdom), maintaining camera and microscope settings identical between various samples, corrected for uneven illumination and background using software functions, and processed using classification and measuring routines in Image-Pro Plus software.

#### Scoring of plasticity in myofibers

A viable nucleic acid stain such as Syto 13 or Hoechst 33342 (Molecular Probes) was routinely used in cultures to visualize and score the myofibers. The quiescent or inactive myofiber nuclei were aligned along the fiber (see Figures 1 and 7D), and the cell did not show any cytoplasmic extensions from the axis. The nuclei in active fibers moved along the axis of the fiber to form aggregations that were localized either in the middle or towards the end of the cell (see Figure 3A, 3B, and 3G). In most cases, this resulted in formation of localised cytoplasmic protrusions in the vicinity of the nuclei. Myofibers were classified and counted based on the above criteria.

#### Taxol inhibition assay.

Dissociated myofibers were plated in medium containing taxol (2 μM; Sigma). Parallel control cultures were incubated in vehicle (DMSO) in a similar way. The cultures were fixed at 48 h after treatment and processed for tubulin immunofluorescence or in situ hybridisation.

#### Functional assay for Msx-1 using morpholino antisense oligonucleotides

Morpholino-based antisense oligonucleotides of 25 oligomere were prepared to target the translation initiation site of axolotl *Msx1* gene (5′-CGGTCTGCATCCTCTGCTTGCTTAG-3′) by Gene Tools Inc. (Corvallis, Oregon, United States). Invert control oligos of *Msx1* (5′-GATTGCTTCGTCTCCTAGCTCTGGC-3′) and standard control oligos (5′-CCTCTTACCTCAGTTACAATTTATA-3′) from the supplier were used as controls. 3′-Biotin-end-labelled standard control oligos were used for evaluating the uptake of morpholino oligonucleotides by myofibers. Oligo stock solutions were prepared according to guidelines from the manufacturer and stored at 4 °C. The morpholino oligos were added at a concentration of 10 μM to the dissociation cocktail and the myofibers were dissociated as described. After purification of myofibers, fresh morpholino oligos were added to the culture medium. After sequential washes to remove any adherent morpholino (McKeon et al. 2001), cultures were fixed at 48 h after treatment, prior to analysis.

#### Detection of morpholino uptake by immunofluorescence.

Tyramide signal amplification (PerkinElmer Life Sciences Inc., Wellesley, Massachusetts, United States) coupled with enzyme-linked immunofluorescence (ELF97, Molecular Probes) was employed to localize the uptake of morpholino oligos in cultured myofibers. Myofiber cultures were fixed at 48 h in 0.5% PFA containing 0.05% glutaraldehyde for 15 min on ice. The fixative was replaced with freshly made 0.1% NaBH_4_ solution and incubated for 5 min. The manufacturer's protocol was employed for TSA amplification with the ELF97 modification. The samples were developed in ELF reaction buffer under fluorescence microscope for 10–20 s and images were acquired using a cooled digital camera.

#### In situ hybridisation

The axolotl *Msx1* cDNA (a kind gift from H. Ide, Tohoku University, Sendai, Japan) was cloned into Bluescribe vector (Stratagene, La Jolla, California, United States), and probes were generated as described elsewhere (Koshiba et al. 1998). A 0.7 kb axolotl EF-1α fragment (kindly provided by D. Gardiner and S. Bryant, University of California, Irvine, United States) was cloned into PCR II vector (Invitrogen, Carlsbad, California, United States) and linearised with XhoI (antisense), and a riboprobe was generated with SP6 RNA polymerase. Newt *Rad* (*NRad*, a gift from K. Yoshizato, Hiroshima University, Hiroshima, Japan) probe was generated from a fragment of approximately 400 bp from Bluescribe vector after linearising with either HindIII (antisense) or EcoRI (sense), and riboprobes were synthesized using T3 and T7 RNA polymerase respectively (Shimizu-Nishikawa et al. 2001). Axolotl *EF1α* and *NRad* probes were used as positive controls, while neomycin (Cash et al. 1998), *NRad* sense, and *Msx1* sense probes served as negative controls. For in situ hybridisation, the myofiber cultures were incubated in BDM (4 mM), fixed in chilled 1% glutaraldehyde for 15 min, postfixed in 4% PFA, and washed in 0.3% PBT. In situ hybridisation was essentially as described elsewhere (Kumar et al. 2000), with minor modifications in the hybridisation temperature.

#### Antibodies and immunofluorescence

Myofiber cultures were routinely fixed in ice-cold 0.5% PFA containing 0.05% glutaraldehyde for 10 min on ice. For β-tubulin staining, 5 μM Taxol (Sigma) was incorporated into the fixative. After fixation, the culture was treated with freshly prepared 0.1% NaBH_4_ for 5 min to reduce nonspecific fluorescence. The samples were post-fixed in ice-cold methanol at −20 °C for 10 min, washed three to four times in 0.3% PBT, and blocked in PBT containing 10% goat serum. The primary antibodies were to MHC and titin, and BrdU monoclonal antibody and rabbit polyclonal antibodies to phosphohistone H3, were all as described elsewhere (Tanaka et al. 1997; Kumar et al. 2000; Velloso et al. 2000; Bettencourt-Dias et al. 2003). For localisation of β-tubulin, the culture was fixed and washed overnight in 0.3% PBT and incubated with mouse monoclonal β-tubulin antibody (1:100; clone TUB 2.1; Sigma) overnight at 4 °C. The samples were washed extensively in GS/PBT and incubated in TR-conjugated goat anti-mouse antibody (1 μg/ml; Molecular Probes). The nuclei were counterstained with Hoechst 33258 (2 μg/ml). A rabbit polyclonal antibody raised against the full-length mouse Msx1 homeoprotein was used to detect expression of Msx1 protein (BAbCO, Richmond, California, United States). When a full-length expression construct of axolotl *Msx1* was expressed in mouse cells by transient transfection, the antibody gave strong and specific staining of nuclei in transfected cells (Figure S2). The samples were fixed and processed as before and incubated with Msx1 antibody (1:1000) overnight at 4 °C. After several washes, the cultures were incubated with FITC-conjugated goat anti-rabbit antibodies (1:100; DakoCytomation, Cambridgeshire, United Kingdom), and the nuclei were counterstained with Hoechst. A control rabbit polyclonal antibody was processed in parallel to obtain a baseline value for quantitative fluorescence measurements on immunostained nuclei.

The myofiber cultures stained for β-tubulin, MHC, or titin, or cultures injected with TR–dextran were observed under confocal laser scanning microscope (Leica). The images were acquired as *z*-stacks, and composite maximum projection images were generated through Leica software. Samples stained for Msx1 protein were observed under a Zeiss Axioplan microscope and images were acquired with an Axiocam digital camera. The fluorescence intensity in myofiber nuclei was measured in Axiovision software (Zeiss), and the data were analysed by one-way analysis of variance (ANOVA) followed by multiple range test using Instat (Graphpad Software Inc., San Diego, California, United States).

## Supporting Information

Figure S1 Live Striated Myofiber Dissociated from the Limb of a Larval Salamander The myonuclei incorporate Syto13 live nuclear stain. The myofiber was observed with VAREL optics at 24 h after plating. Scale bar, 100 μm.
(4.1 MB TIF).

Figure S2 Expression of Newt *Msx1* in Mouse PS Cells by Transient Transfection Nuclear localisation of Msx1 protein (green) was detected with a rabbit polyclonal antibody generated against the full-length mouse Msx1 homeoprotein.
(5.6 MB TIF).

Video S1 Time-Lapse Video Analysis of Nuclear Migration in a Myofiber Time-lapse sequence was begun 6 h after plating of the myofiber on to a culture dish. The images were taken at 6 min intervals under 32× VAREL objective magnification.
(110 KB AVI).

## Abbreviations

AMEM: amphibian minimum essential medium
ANOVA: analysis of variance
BDM: 2,3-butanedionne monoxime
BrdU: 5-deoxy-2′-bromouridine
MHC: myosin heavy chain
PFA: paraformaldehyde
TR: Texas red-conjugated dextran
VAREL: variable relief contrast
