# Supplementary figures and images for: The Regenerative Plasticity of Isolated Urodele Myofibers and Its Dependence on Msx1
Source: PLoS Biol. 2004 Aug 17;2(8):e218. doi: 10.1371/journal.pbio.0020218 (PMC509293; doi:10.1371/journal.pbio.0020218)

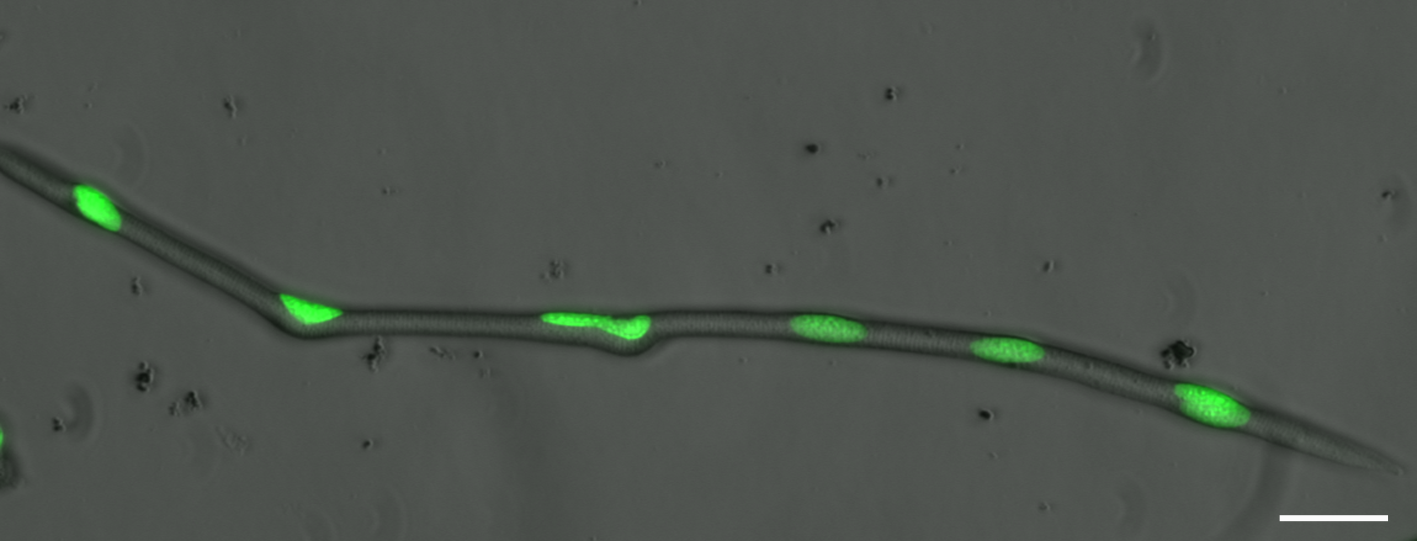

Supplement: Figure S1 — The myonuclei incorporate Syto13 live nuclear stain. The myofiber was observed with VAREL optics at 24 h after plating. Scale bar, 100 μm. (4.1 MB TIF). [file pbio.0020218.sg001.tif]

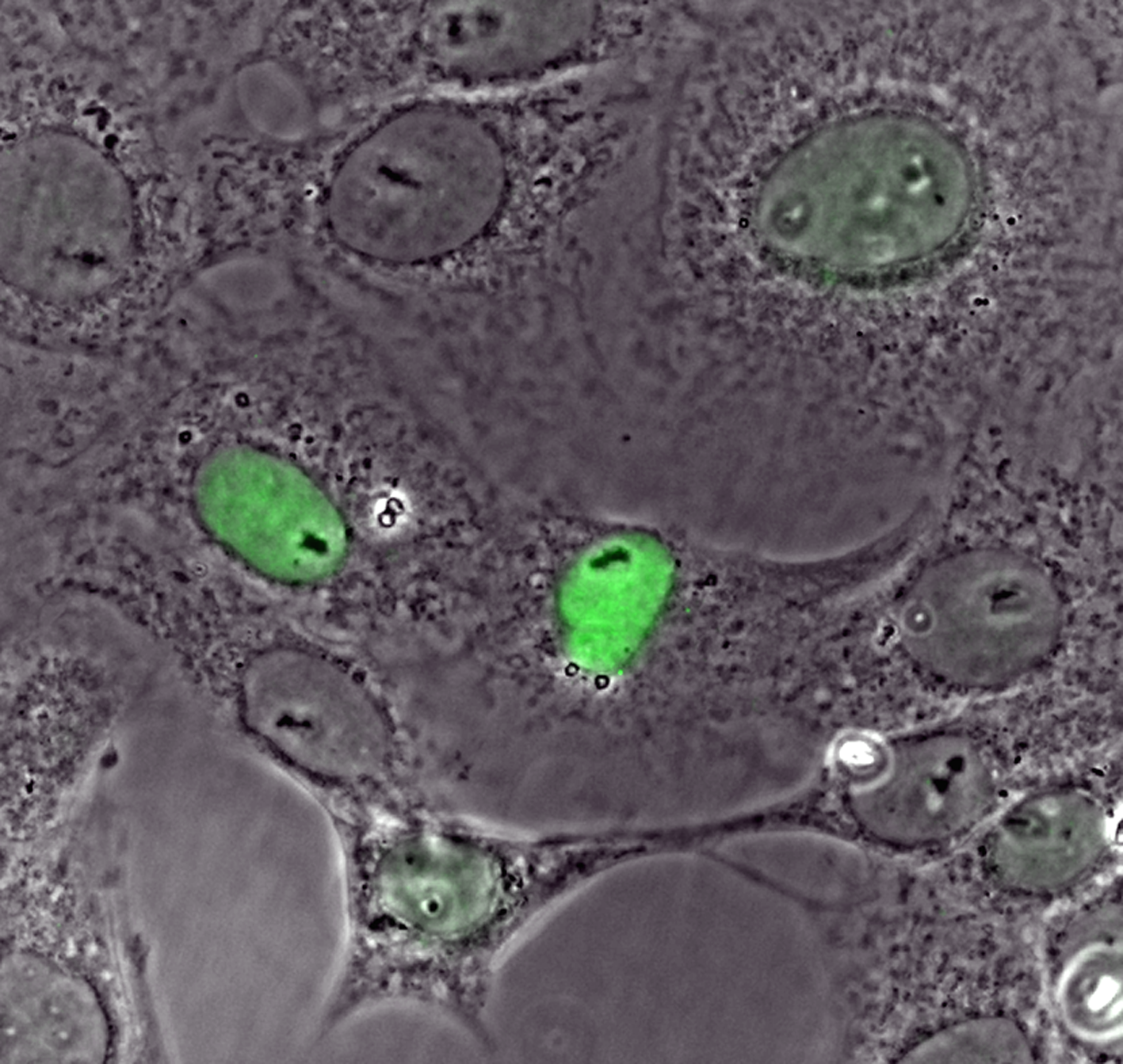

Supplement: Figure S2 — Nuclear localisation of Msx1 protein (green) was detected with a rabbit polyclonal antibody generated against the full-length mouse Msx1 homeoprotein. (5.6 MB TIF). [file pbio.0020218.sg002.tif]
